# Supplementary material for: Identification of photoperiod-induced specific miRNAs in the adrenal glands of Sunite sheep (Ovis aries)
Source: Front Vet Sci. 2022 Jul 22;9:888207. doi: 10.3389/fvets.2022.888207 (PMC9354845; doi:10.3389/fvets.2022.888207)
Supplement: Supplementary file 1 [file Data_Sheet_1.ZIP › Supplementary file/miRDeep2 file.zip!/miRDeep2 file/Know_Structure/SPLP42A3/oar-mir-16b.pdf]

miRBase precursor : oar-mir-16b
Total read count : 20780
oar-miR-16b read count : 20754
remaining reads : 26

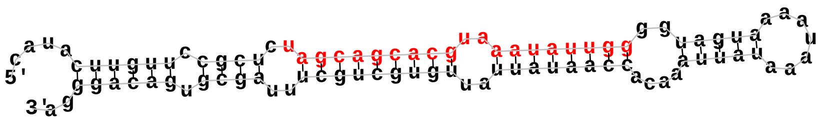

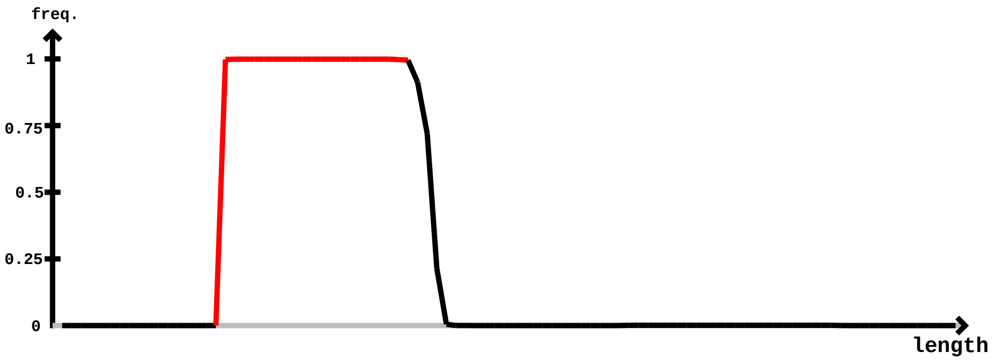


```
                oar-miR-16b
5'- cauacuuguuccgcucuagcagcacguaaauauuggggguaguaaaauaaauauuaaacaccaauauuauugugcugcuuuagcgugacaggga   -3'    exp
    ....((((((.((((..(((((((((..((((((((..(((((.......)))))....))))))))..)))))))))..)))).))))))..   reads      mm         sample
    ...............cuagcagcacguaaauauugg.........................................................   1          0          seq
    ...............cuagcagcacguaaauauuggggu......................................................   1          0          seq
    ................uagcagcacguaaauauu...........................................................   37         0          seq
    ................uagcagcacguaaauauug..........................................................   39         0          seq
    ................uagcagcacguaaauauugg.........................................................   1724       0          seq
    ................uagcagcacguaaauauuggA........................................................   3792       1          seq
    ................uagcagcacguaaauauuggg........................................................   203        0          seq
    ................uagcagcacguaaauauugggg.......................................................   843        0          seq
    ................uagcagcacguaaauauuggAg.......................................................   9655       1          seq
    ................uagcagcacguaaauauuggggu......................................................   1819       0          seq
    ................uagcagcacguaaauauuggAgu......................................................   2524       1          seq
    ................uagcagcacguaaauauuggggua.....................................................   94         0          seq
    ................uagcagcacguaaauauugggguag....................................................   1          0          seq
    ................uagcagcacguaaauauugggguagu...................................................   5          0          seq
    .................agcagcacguaaauauugg.........................................................   1          0          seq
    .................agcagcacguaaauauuggA........................................................   2          1          seq
    .................agcagcacguaaauauugggg.......................................................   2          0          seq
    .................agcagcacguaaauauuggAg.......................................................   10         1          seq
    .................agcagcacguaaauauuggAgu......................................................   1          1          seq
    ..................gcagcacguaaauauuggAg.......................................................   3          1          seq
    ..................gcagcacguaaauauuggAgu......................................................   2          1          seq
    .........................................................caccaauauuauugugcugcuuu.............   2          0          seq
    ..........................................................accaauauuauugugcugcuuu.............   13         0          seq
    ..........................................................accaauauuauugugcugcuuua............   4          0          seq
    ...........................................................ccaauauuauugugcugcuuu.............   2          0          seq
```
